# Self-perceived oral health in hemato-oncological patients and the relation to quality of life

**DOI:** 10.1007/s00520-024-08849-w

**Published:** 2024-09-07

**Authors:** Alexa M.G.A. Laheij, Linda M. Dillen, Erfan Nur, Judith E. Raber-Durlacher

**Affiliations:** 1 Department of Oral Medicine, Academic Centre for Dentistry (ACTA), University of Amsterdam and Vrije Universiteit Amsterdam, Gustav Mahlerlaan 3004, 1081 LA Amsterdam, The Netherlands; 2 Department of Oral Maxillofacial Surgery, Amsterdam UMC, University of Amsterdam, Amsterdam, The Netherlands; 3 Department of Hematology, Amsterdam UMC, University of Amsterdam, Amsterdam, The Netherlands; 4 Sanquin Research and Landsteiner Laboratory, Department of Blood Cell Research, Amsterdam, The Netherlands

**Keywords:** Oral health, Oral health-related quality of life, Hematologic malignancy, EORTC-QLQ-OH15, OHIP-15, Xerostomia

## Abstract

**Purpose:**

To assess the self-reported oral health and oral health-related quality of life of patients diagnosed with hemato-oncological disease.

**Patients and methods:**

Data was collected through a digital questionnaire in collaboration with the Dutch patient organization Hematon. The questionnaires EORTC-QLQ-C30, EORTC-QLQ-OH15, shortened Xerostomia Inventory (XI), and the OHIP-14 were used.

**Results:**

Seven hundred five patients were included (52.5% female, mean age 63.2 ± 10.1). The majority was diagnosed more than 2 years ago (86%) and had received treatment (81%) for their disease. Lymphoma, leukemia, and multiple myeloma were the most frequent malignancies. Chemotherapy alone, chemotherapy in combination with targeted therapy or immunotherapy, and myeloablative chemotherapy followed by autologous stem cell transplantation were the most common treatment modalities. The XI identified that 40.5% met the criteria for xerostomia. Other complaints included mouth soreness and sensitivity, gingival pain and bleeding, problems with teeth or with an ill-fitting denture. Despite reporting oral complaints, most patients experienced a rather good OH-QoL. A high xerostomia score led to a significantly lower OH-QoL. Female gender, history of stem cell transplantation, radiation to head and neck, and multiple daily medication use were significant predictors of xerostomia.

**Conclusion:**

Patients with hematologic malignancies frequently reported a dry mouth and other oral complaints including mouth soreness and sensitivity, gingival pain and bleeding, and problems with teeth. Despite these oral complaints, most patients experienced a relatively good OH-QoL. Future longitudinal studies are needed, and health professionals should have an active role in providing oral supportive care based on patients’ individual needs.

**Supplementary Information:**

The online version contains supplementary material available at 10.1007/s00520-024-08849-w.

## Introduction

In the Netherlands, yearly more than 10,000 patients are newly diagnosed with hematologic malignancies, mostly in adult patients. While watchful waiting is applied in patients with slowly progressing malignancies, most patients with hematologic malignancies are treated with chemotherapy, often in combination with immunotherapy or radiotherapy. In selected high-risk diseases, patients undergo chemotherapy followed by an autologous (stem cells from the patient) or allogeneic (stem cells from a donor) stem cell transplantation (SCT) [1, 2]. Autologous SCT can be administered in patients with multiple myeloma, (non)Hodgkin lymphoma, and acute myeloid leukemia, while allogeneic SCT can be administered for acute leukemia, (non)Hodgkin lymphoma, and myelodysplastic syndrome.

Patients treated for hematologic malignancies may experience treatment-induced complications of the oral cavity and peri-oral tissues. Oral mucositis is one of the most frequent acute complications of treatment, affecting quality of life negatively [3]. It is characterized by erythema, ulceration, pain, difficulty eating, and higher need for pain medication. Other acute complications of hemato-oncologic therapies include xerostomia (feeling of a dry mouth), hyposalivation (decreased saliva production), oral infections, and taste changes [4]. In most cases, these complications resolve within weeks or months following the completion of chemotherapy, but may persist and become chronic in some patients.

All SCT patients may also experience late oral complications such as xerostomia, hyposalivation, higher caries activity, and taste changes, possibly leading to lower quality of life. Moreover, patients that received an allogeneic SCT may develop oral chronic graft-versus-host disease [5]. These patients may suffer from oral mucosal pain and ulceration, mucosal sensitivity to certain foods and liquids, dry mouth, loss of taste, and more rarely from a decreased mouth opening. Patients with a history of oral chronic graft-versus-host disease are at increased risk for oral cancer [6]. Salivary gland dysfunction leads to a higher risk of caries and fungal infection, as protective properties of saliva are less present in the oral cavity. Several drugs used in the treatment of hemato-oncologic patients cause dry mouth and thereby also contribute to this problem [7].

Patients with multiple myeloma treated with bisphosphonates as antiresorptive medications are at increased risk of developing medication-related osteonecrosis of the jaw. This is characterized by exposed bone and can be accompanied by purulent discharge, fistula, erythema, swelling, and pain. Risk factors include an invasive dental procedure, advanced periodontitis, smoking, older age, and diabetes mellitus [8].

Patients with hematologic malignancies are also increasingly treated with targeted therapies and immunotherapies often in combination with one or more chemotherapeutic agents. Targeted therapies inhibit the growth of malignant cells by targeting specific molecular targets that are essential for tumor growth. Although these therapies are more specifically directed to interfere with cancer growth than classical cytotoxic oncological therapy, there are still adverse side effects, also in the oral cavity. Patients may develop oral mucosal lichenoid changes and ulceration, xerostomia/hyposalivation, and taste changes [7].

Studies on long-term oral complications of therapies for hematologic malignancies are scarce. Since the number of patients with hematologic malignancies is increasing due to both increased incidence and improved survival [9], more patients will possibly suffer from acute and chronic oral complications after treatment. It is not clear to what extent these patients report these oral complications. Therefore, the aim of our study was to examine the self-reported oral health and oral health-related quality of life of patients diagnosed with hemato-oncological disease. This will contribute to obtaining a better profile of oral complications in this patient group, providing opportunities for tailoring oral care.

## Materials and methods

This study was approved by the institutional review board of Amsterdam University Medical Centre (registration number W21_272 no. 21.299) and performed according to the Declaration of Helsinki [10]. All adult participants provided written informed consent.

### Study population and data collection

Data were collected in collaboration with Hematon, the Dutch association of patients diagnosed with hemato-oncological disease, that has about 5700 members. All members were invited to participate through the newsletter and the online channels of the organization. The study survey was made available digitally through these channels. Data collection was completely anonymous. Data collected within the first 3 months after publication of the newsletter were used in this study (until October 2021). Patients who did not at least complete the general questionnaire and OHIP-14-NL were excluded from analyses.

### Questionnaires

First general questions were asked about patient characteristics, medical diagnosis, treatment (history), and dental status. The participants were then asked to complete validated questionnaires regarding quality of life (EORTC QLQ-C30 version 3.0) [11], oral health-related quality of life (EORTC QLQ-OH15) [12], the impact of oral symptoms on quality of life (OHIP-14) [13], and xerostomia (complaints of dry mouth) (shortened XI-NL) [14]. EORTC Quality of Life Group approved of using the EORTC questionnaires.

### Data analyses

Data were analyzed using SPSS Statistics version 28.0.1.1 (IBM). Data are presented as mean with SD. Results were analyzed using descriptive statistics. Differences in groups regarding the presence/absence of xerostomia were analyzed using the Chi-square test. Correlations were calculated using the Pearson correlation coefficient. Predictors for xerostomia scores were calculated by linear regression analysis using the enter method. A *p* value < 0.05 was considered statistically significant.

## Results

In total, 764 (partial) responses were recorded, yielding a response rate of 13.4%. Three respondents had actively declined informed consent, and 41 responses were excluded due to insufficient survey progress. Reasons why not to partake in or complete the survey included the following: never having experienced problems with his/her dentition or having no recent oral problems and therefore rendering their response as useless. An additional 15 responses were excluded due to concerning patients affiliated with patient association, Hematon, for other reasons than being diagnosed with a hematologic malignancy themselves. The final sample size consisted of 705 patients. Of the 705 patients, all completed the questions regarding oral hygiene, and 571 about the treatment (history). Concerning the validated questionnaires, 696 patients completed the EORTC QLQ-C30, 695 the EORTC OH-15, 694 the shortened XI, and 545 the OHIP-14.

### Patient characteristics

All patients were previously treated for hemato-oncological diseases; 52.5% were female. The mean age was 63.2 (± 10.1) years, ranging from 21 to 86 years. Most participants were diagnosed with lymphoma (36.0%), followed by leukemia (27.1%) and multiple myeloma (19.9%). The majority of them were diagnosed more than 2 years ago (87%) and had received or were receiving treatment (81%) at the time of the completion of this survey. Of the previously treated patients, 38.9% had been treated within 12 months prior to the survey, whereas 45.9% had received their last treatment more than 2 years ago (Appendix Table 5). Chemotherapy, often in combination with targeted therapy or immunotherapy, was the most common treatment modality, followed by myeloablative chemotherapy and autologous SCT. Approximately, half (49.0%) of treated patients underwent more than one line of treatment.

A total of 223 (31.6%) patients received one or multiple SCTs, of which in 221 patients, hemato-oncologic disease formed the rationale for SCT treatment. Most SCT patients received an autologous SCT (Appendix Table 5). In total, 14.8% of patients received radiation to the head and neck area. Patients varied in daily prescription medication use, with 29.6% of the patients using four or more different prescribed drugs on a chronic basis. Part of the patients (15.7%) had also received treatment for other forms of cancer, including head and neck cancer in 26 patients (3.7%).

While 307 (43.5%) patients indicated never to have smoked, or used drugs, and never or rarely drunk alcohol, 312 (44.3%) had a history of smoking (Table 1). Majority of the patients (62.1%) had at least completed a bachelor’s degree.

**Table 1:** Patient characteristics

Variables	Groups	Mean	SD	Minimum-maximum
Age		63.2 years	10.1	21 – 86
			***N***	**%**
Gender	Male		335	47.5
	Female		370	52.5
Hematologic malignancy (diagnosis)	Lymphoma		254	36.0
	*Non-Hodgkin lymphoma*		197	77.6
	*Hodgkin lymphoma*		45	17.7
	*Hairy cellleukemia*		5	2.0
	*Not known*		7	2.8
	Leukemia		191	27.1
	*Acute*		53	27.7
	*Chronic*		132	69.1
	*Not known*		1	0.5
	Multiple myeloma/Kahler’s disease		140	19.9
	Myelodysplastic syndrome (MDS)		21	3.0
	Other hematologic malignancy		81	11.5
	Multiple diagnoses		20	2.8
	Missing		3	0.4
	Not known		-	-
Daily prescription medication use	No (current) use of daily medication		167	23.7
	Use of 1 daily medicine		132	18.7
	Use of 2 or 3 different daily medications		197	27.9
	Use of 4 or more different daily medications		209	29.6
Smoking habits (daily)	Yes, but something other than cigarettes (e.g., cigars, e-cigarettes)		6	0.9
	≤ 1 cigarette		4	0.6
	2–5 cigarettes		5	0.7
	5–10 cigarettes		6	0.9
	≥ 10 cigarettes		12	1.7
	Quit smoking		355	50.3
	Never smoked		317	45.0
Alcohol use (daily)	≤ 2 glasses		367	52.1
	> 2 glasses		30	4.3
	Quit drinking alcohol		219	31.1
	Never drank alcohol		89	12.6

### Dental check-up visits, oral hygiene habits, and number of teeth

Most patients (88.1%) went for a dental check-up at least yearly and brushed their teeth daily with a fluoride toothpaste. Of these, 82% performed interdental cleaning multiple times a week. (Appendix Table 6). Patients reported having an average of 23 (± 7) teeth present in the oral cavity. Thirty patients (4.3%) were edentulous. Almost one out of five patients who received cancer treatment (17.7%) had been advised by an oncology health professional to temporarily postpone appointments with a dentist or dental hygienist; these were mostly allogeneic SCT recipients (33%).

### Global quality of life

In general, patients experienced a good quality of life and a proper level of functioning (Appendix Fig. 2). Regarding global health symptoms, patients most frequently experienced fatigue, insomnia, and pain. Both items covering overall health and overall quality of life in the prior week were given a score of more than 4 by 65.5% (*n*=462) of patients (Appendix Fig. 3).

### Oral health-related quality of life assessed by the QLQ-OH15 questionnaire

**Fig. 1:**
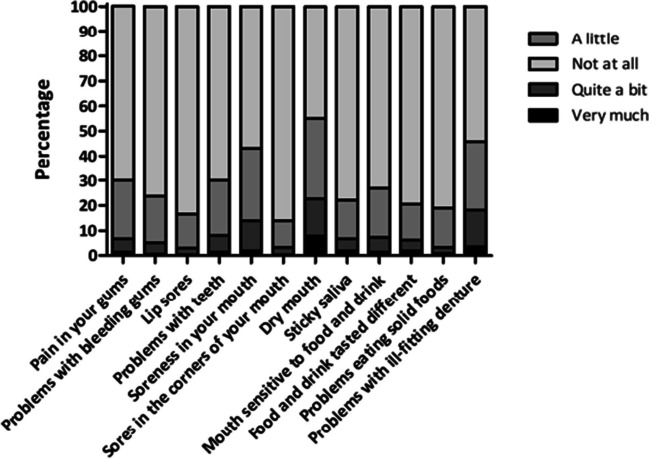
QLQ-OH15. Scores per question, *N*=695

Patients reported a mean score of 88.1 (±12.4, range 12.5–100.0) on the QLQ-OH15 QoL scale, what qualifies as good. However, more than half of all patients reported having symptoms of dry mouth in the past week (54.9%), of which 7.8% experienced severe xerostomia (Fig. 1).

Other common symptoms were mouth soreness (43.1%), problems with teeth (30.1%), and pain in gums (29.8%). Nearly half (45.8%) of the denture-wearing patients (*n*=83) experienced problems with an ill-fitting denture. Most patients who experienced symptoms reported these as “little” or “quite a bit” present.

### Xerostomia

As much as 40.5% of patients met the criteria for xerostomia as assessed by the shortened Xerostomia Inventory (Appendix Table 7). The distribution of the presence of xerostomia between different variables is shown in Table 2. Xerostomia was most common in patients who had undergone allogeneic SCT, with as many as 62.5% of these patients qualifying as having xerostomia. Prevalence of xerostomia was significantly associated with, female gender, number of daily prescription medication, history of allogeneic SCT, and whether radiation to the head and neck was received was statistically significant (Table 2, *p *< .05).

**Table 2:** Xerostomia prevalence

Variable	*N*	Xerostomia		*p* value
		No %	Yes %	
**Largest distinct hemato-oncological treatment groups (*N*=516)**				.033*
No treatment (including watch-and-wait)	133	59.4	**40.6**	
Chemotherapy and targeted/immunotherapy	93	65.6	**34.4**	
Chemotherapy	90	58.9	**41.1**	
Autologous SCT	81	60.5	**39.5**	
Allogeneic SCT	64	37.5	**62.5**	
Chemotherapy and radiotherapy	33	54.5	**45.5**	
Targeted/immunotherapy	22	54.5	**45.5**	
**Gender (*N*=694)**				<.001*
Male	332	68.4	**31.6**	
Female	362	50.0	**50.0**	
**Daily prescription medication use (*N*=694)**				<.001*
No (current) use of daily medication	163	71.8	**28.2**	
Use of 1 daily medicine	130	65.4	**34.6**	
Use of 2 or 3 different daily medications	196	54.1	**45.9**	
Use of 4 or more different daily medications	205	48.8	**51.2**	
**Age groups (*N*=694)**				.070
Under 50	48	64.6	**35.4**	
50 up to and including 59	167	54.5	**45.5**	
60 up to and including 69	286	55.6	**44.4**	
70 and up	193	65.8	**34.2**	
**Most recent hemato-oncological treatment (*N*=694)**				.089
No hemato-oncological treatment received	125	60.8	**39.2**	
Within the past month	113	57.5	**42.5**	
1–3 months ago	35	60.0	**40.0**	
3–12 months ago	70	74.3	**25.7**	
1–2 years ago	84	60.7	**39.3**	
More than 2 years ago	258	54.3	**45.7**	
Missing or patient does not know	9			
**History of SCT (*N*=694)**				<.001*
No SCT	464	61.2	**38.8**	
Yes, 1 or more autologous SCT	133	63.9	**36.1**	
Yes, 1 or more allogeneic SCT	65	33.8	**66.2**	
Yes, both autologous and allogeneic SCT	18	50.0	**50.0**	
Missing	14			
**Received radiation to head and neck (*N*=694)**				.002*
Yes	102	45.1	**54.9**	
No	580	61.6	**38.4**	
Patient does not know or missing	12			
**Received treatment for other forms of cancer (*N*=694)**				.406
Yes, within the head and neck area, additionally also outside the head and neck area	26	50.0	**50.0**	
Yes, exclusively outside the head and neck area	78	64.1	**35.9**	
No	586	58.2	**41.8**	
Missing	4			
**Smoking habits (*N*=694)**				.501
Less than 10 cigarettes daily	15	40.0	**60.0**	
10 or more cigarettes daily	11	54.5	**45.5**	
Quit smoking	351	59.3	**40.7**	
Never smoked	311	59.5	**40.5**	

*p<.05

All categories that were significantly associated to xerostomia were added as independent variables to the linear regression model with the xerostomia score as dependent variable. Results are shown in Table 3. Women, patients that use more daily prescribed medications, patients that were previously irradiated to the head and neck area, and patients with history of allogeneic SCT reported more xerostomia.

**Table 3:** Predictors for xerostomia score

Predictors	Unstandardized coefficients B	*p* value
Largest distinct hemato-oncological treatment groups	−0.018	.699
History of SCT	.161	.024*
Female gender	1.323	<.001*
Received radiation to head and neck	−.713	.016*
Daily prescription medication use	.466	<.001*

*p<.05

### Oral health-related quality of life assessed by the OHIP-14 questionnaire

Oral health-related quality of life assessed by the OHIP-14 showed that the majority of patients (77.3%) had experienced recent oral health-related complaints; however, these problems did not occur on a regular basis as the scores per domain were generally low. When focusing on the highest reported domain score per patient, for most patients (83.3%), this entailed a score of 4 or less (graded on a scale ranging from 0 to 8 with 0 being no complaints and 8 being severe complaints). Physical pain (68.5% of patients) and psychological discomfort (48.4% of patients) were the most commonly reported problem areas, and simultaneously these areas were reported as most affected by most patients (Appendix Table 8).

### Relation between oral health-related quality of life and xerostomia

There was a moderate negative correlation between xerostomia and the oral health-related quality of life subscale score (QLQ-OH15). More severe symptoms of dry mouth were related to a worse oral health-related quality of life. There was a low positive correlation between xerostomia score and the OHIP-14 total score. More severe symptoms of dry mouth were related to more frequent oral health-related problems. And there was a low negative correlation between xerostomia score and global health-related quality of life scale score. More severe dry mouth symptoms were related to lower experienced quality of life (Table 4).

**Table 4:** Pearson correlation coefficients for xerostomia and oral health-related and global quality of life (*N*=694)

	Xerostomia score
Eight-item OH-QoL scale (QLQ-OH15)	−.588**
OHIP-14 total score	.476**
Global health status/quality of life (QLQ-C30)	−.304**

**p value < .01

## Discussion

This study shows that a substantial portion of patients treated for hemato-oncological disease reported serious complaints of having a dry mouth. More than half of the patients reported having a dry mouth over the last week with severities ranging from mild to severe. Of these, 40.5% met the criteria for xerostomia, and a higher xerostomia score led to a significantly lower oral health-related quality of life. Female gender, history of allogeneic SCT, radiation to head and neck, and multiple daily medication use were significant predictors of xerostomia.

Other oral complaints included mouth soreness and sensitivity, gingival pain and bleeding, problems with teeth, and problems with an ill-fitting denture (if applicable), which patients mostly assessed as mild. Despite reporting recent oral complaints, most patients experienced a rather good oral health-related quality of life. This notion is further supported by patients mostly reporting low OHIP-14 domain- and total scores: the majority of respondents encountered problems related to their oral health, though not on a regular basis, experiencing their oral health-related quality of life as good.

A potential explanation for the relatively good oral health-related quality of life could be that patients with often life-threatening hematologic malignancies might have an altered perception of the relatively milder oral complaints on their quality of life after having experienced extremely painful and bothersome oral and non-oral complications (i.e., severe oral mucositis) associated to the treatment. Another explanation might be in the specifics of the study cohort. Members of Hematon, especially those responding to this survey might be higher educated and better taking care of themselves.

The OHIP-14 results showed that in our study participants, the most common and reoccurring problem areas were physical pain and psychological discomfort. With respect to physical pain, this is in line with the findings of Stolze et al. [15], who performed a systematic review on the impact of hematologic malignancies and their treatment on OH-QoL assessed by the OHIP-14. In most of the studies that met the inclusion criteria, the domain physical pain was frequently given a high score [16–18]. The patients included in these studies were mostly in the early phase of their treatment, whereas the time since treatment of the patients in our study varied, with a relatively large group that had completed their cancer treatment more than 2 years ago. This points to the fact that patients may experience persistent oral pain and problems with eating even after oral mucositis has resolved, warranting further investigation.

Significant predictors for xerostomia were female gender, use of multiple prescribed daily medications, history of allogeneic SCT, and exposure to head and neck radiation therapy. A large epidemiological Swedish study involving the general population found a strong relationship between the number of medications taken and the presence of xerostomia. The probability of developing xerostomia increased with age and was also higher in women [19, 20]. Another Swedish study confirmed these results regarding gender and number of medications taken [21]. Not only the quantity, but also the type of medication and its therapeutic effect can impact oral health and dry mouth symptoms. In addition, many patients reported treatment with targeted and/or immunotherapies, which may be used for a prolonged period of time and are often associated with xerostomia [7].

Xerostomia prevalence in our group seems higher compared to other studies. Especially, since the definition of xerostomia in our study was stricter than in other studies. In our study, xerostomia was only indicated if patients scored at least “sometimes” at least three questions from the shortened XI, others included forms of very mild xerostomia complaints as well [20–22].

Curative radiotherapy within the head and neck area is known to damage salivary glands and diminish salivary flow [4]. Approximately, one in seven patients reported having had radiotherapy within this area. However, most patients with hematologic malignancies do not receive high-dose radiotherapy to the head and neck region. As a portion of patients (15.7%) also received treatment for other non-hematologic malignancies, including head and neck cancer in 26 patients (3.7%), this could be a plausible explanation for the high incidence of reporting head and neck radiotherapy. However, these groups did not completely overlap, indicating that the data on head/neck radiotherapy could be less valid. It is understandable that patients could have difficulties classifying their radiotherapy.

Xerostomia could be a factor as to why many denture-wearing patients in our study experience problems. While xerostomia is not always associated with hyposalivation, it is advisable that dentists assess the stimulated and unstimulated whole salivary flow in order to determine whether preventative measures in dentate and edentate patients are indicated. Patients suffering from a dry mouth may benefit from oral moistening products and in some cases from pilocarpine, a muscarinic cholinergic agonist [22]. Moreover, patients with hyposalivation have an increased risk of mucosal infection, particularly candidiasis, which may cause oral pain and discomfort.

In our study, we asked for potential concerns about visiting dental professionals expressed by the oncology team. Almost one out of five patients who received cancer treatment, allogeneic SCT in particular, had been advised by an oncology health professional to temporarily postpone appointments with a dentist or dental hygienist. This advice is given out of fear that dental treatment may induce bacteremia and systemic infectious complications in patients that are immunocompromised for several months, or even years after SCT. This situation is predominantly present in allogeneic SCT recipients, not in autologous SCT recipients. However, this risk is not present in case of preventive dental interventions aimed at keeping gingival inflammation and the microbial load low and prevent rapidly progressive dental caries in patients with salivary gland dysfunction. A dental evaluation preferably before the start of therapy is considered as the standard of care in patients with hematologic malignancies [23]. Preventative measures before and during therapy may reduce infectious complications originating from the oral cavity [24]. Invasive dental procedures should be postponed during phases of neutropenia and thrombocytopenia as much as possible and when unavoidable should be performed in close consultation with the hematologist.

The response rate of our study (13.4%) is comparable with other studies using questionnaires to evaluate oral health [25]. Nevertheless, with this response rate, patients are represented by a relatively small portion presumably affecting external validity negatively. We cannot exclude that more patients with oral complaints were willing to participate than those without any oral complaints. Or that the patients that are more aware of their health and are more willing to express their opinion are overrepresented. In addition, some patients may have declined participation because of difficulties to complete the digital questionnaires.

Differences in self-perceived oral health and QoL may be different between patients with different treatment trajectories. For instance, SCT increases risk for oral complaints more strongly than other hemato-oncological treatments. Notably, patients reported 55 distinct treatment trajectories illustrating the challenge in defining these patients and knowing what to look for regarding oral health complications. That is why, despite the relatively large sample size, more in depth analyses of our data are not possible. With future individualized treatment plans, this will become even more difficult. Another limitation was that we could not differentiate further between different diagnoses since we did not have access to more detailed information.

The results indicate that a large portion of the patients experience oral problems, throughout oncology therapy and thereafter. Especially xerostomia, that negatively affected quality of life, is the domain of complaints. Hematologists and nurses should be sensitive to identify oral health problems and refer the patients to the oral health team for the proper support. The oral health team should consider the possible acute and long-term negative effects of cancer treatment on oral health and should regularly ask for symptoms and evaluate oral health in order to provide interventions aimed to alleviate complaints and prevent complications.

In conclusion, patients with hematologic malignancies frequently reported having a dry mouth and other oral complaints including mouth soreness and sensitivity, gingival pain and bleeding, and problems with teeth. Despite of having these oral complaints, most patients experienced a rather good OH-QoL. Future longitudinal studies are needed, and medical and dental health professionals should have an active role in providing oral supportive care based on patients’ individual needs.

## Supplementary information

ESM 1 (DOCX 114 kb)

## Figures and Tables

**Fig. 2:**
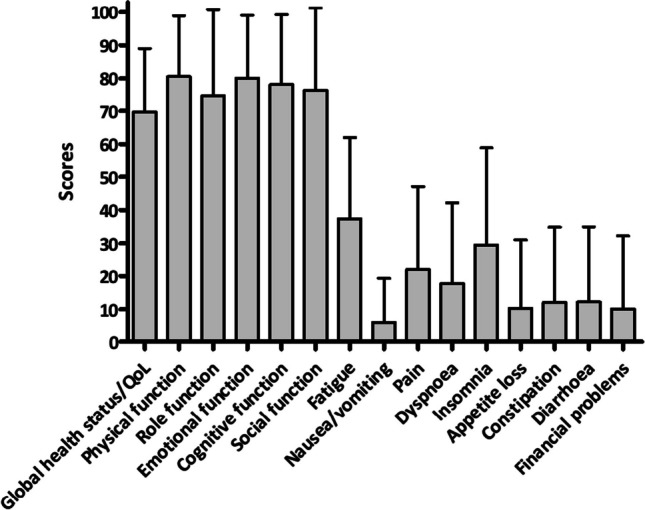
Mean scores EORTC QLQ-C30 ± 1SD, scales and items (*N*=696)

**Fig. 3:**
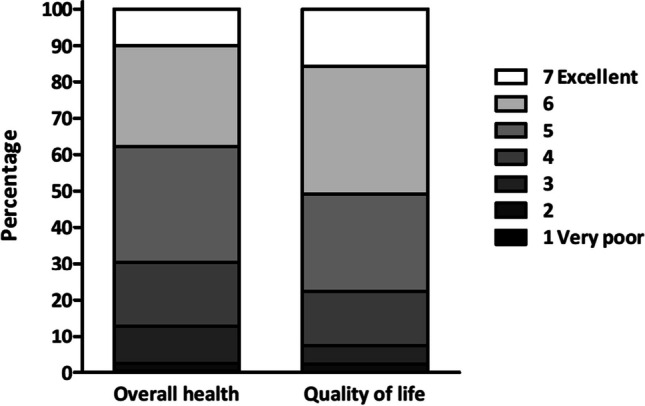
Percentages of the rated global health status and quality of life (EORTC QLQ-C30) (*N*=696)

## Appendix

**Table 5:** Hemato-oncological treatment history, categorized per treatment modality

**(Former) patients** *N =* 705						***N***	**%**
**Received hemato-oncological treatment**	Yes					571	81.0
	No (includes “watch-and-wait” approach)					127	18.0
	Missing					7	1.0
**Time since diagnosis**	0–12 months ago					91	12.2
	2–5 years ago					270	38.7
	> 5 years ago					337	48.3
**Treated patients** (*n*= 571)						***N***	**%**
**Latest hemato-oncological treatment session**	Within past month					116	20.3
	1–3 months ago					36	6.3
	3–12 months ago					70	12.3
	1–2 years ago					85	14.9
	> 2 years ago					262	45.9
	Not known					1	0.2
	Missing					1	0.2
**History of hemato-oncological treatment: occurrence of each treatment modality** (*n* = 571)*****							
Grouped based on **chemotherapy modality**			***N***	**%**		***N***	**%**
	**Chemo-therapy****					**356**	**62.3**
		Chemotherapy, no radiotherapy	258	45.2		142	24.9
					+Targeted/immunotherapy	116	20.3
		Chemotherapy and radiotherapy*******	97	17.0		59	10.3
					+ Targeted/immunotherapy	38	6.7
		Unknown	1	0.2		1	0.2
Grouped based on **SCT modality**	**SCT**					**221**	**38.7**
		Autologous	136	23.8		86	15.1
					+ Targeted/immunotherapy	50	8.8
		Allogeneic	65	11.4		57	10.0
					+ Targeted/immunotherapy	8	1.4
		Both (autologous followed by allogeneic transplantation)	18	3.2		16	2.8
					+ Targeted/immunotherapy	2	0.4
		Unknown	2	0.4		2	0.4
Grouped based on **targeted and/or immunotherapy treatment**	**Targeted therapy and/or immuno-therapy**					**208**	**36.4**
		No chemo-, radio-, or SCT-therapy				24	4.2
Grouped based on **non-specified forms of treatment** (Other than chemo-, radio-, SCT- or targeted/immunotherapy)							
	**Non-specified forms of treatment**					**71**	**12.4**
		Not combined with any specified forms of treatment (chemo-, radio-, SCT- and/or targeted/immunotherapy)				41	7.2

*Half (49.0%) of treated patients underwent more than one type of treatment modality

**Chemotherapy did not include chemotherapy as conditioning regimen for stem cell transplantation

***Radiotherapy did not include radiotherapy as conditioning regimen for stem cell transplantation

**Table 6:** Oral hygiene habits

		*N*	%
Toothbrushing	Two times a day or more	523	74.2
	Once a day	172	24.4
	Multiple times a week	6	0.9
	Once a week or less	4	0.6
Interdental cleaning	Once a day or more	384	54.5
	Multiple times a week	144	20.4
	Once a week or less	132	18.7
	Does not apply due to (partial) dentures	45	6.4
Use of toothpaste with fluoride	Yes	553	78.4
	No	100	14.2
	Patient does not know	52	7.4
Dental check-up	Once a year or more	621	88.1
	Once every 2 or 3 years	21	3.0
	Irregularly	35	5.0
	Only in case of dental emergency	21	3.0
	Never	7	1.0

**Table 7:** Distribution of total scores of the shortened Xerostomia Inventory

Total score	*N*	%
5	202	28.7
6	111	15.7
7	95	13.5
8	90	12.8
9	71	10.1
10	52	7.4
11	25	3.5
12	20	2.8
13	12	1.7
14	8	1.1
15	8	1.1
Missing	11	1.6

A total score ≥ 8 qualifies as xerostomia

**Table 8:** OHIP-14 Highest ranked domain(s) per patient, for patients scoring > 0 on any domain (*N*=545)

Domain	Domain ranked as most affected by patient/highest scoring domain per patient (frequency)*
Functional limitation	52
Physical disability	34
Physical pain	387
Psychological disability	47
Psychological discomfort	174
Social disability	19
Social handicap	17

***Some patients reported multiple equally high scoring domains, which explains a total frequency *>* 545

## References

[1] Nederlandse Kankerregistratie (NKR) IKNL [Netherlands Cancer Registry (NCR) IKNL]. Incidentie per jaar, hematologische maligniteiten [Incidence by year, haematological malignancies] [Internet]. 2023, Feb 3 [2023, Mar 27]. Available from: https://iknl.nl/nkr-cijfers.

[2] DeVita VT Jr, Lawrence TS, Rosenberg SA, Hellman S (2023) DeVita, Hellman, and Rosenberg’s cancer: principles & practice of oncology, Twelfth edn. Lippincott Williams & Wilkins, Philadelphia

[3] Sonis ST (2004) The pathobiology of mucositis. Nat Rev Cancer 4(4):277–284 15057287 10.1038/nrc1318

[4] Epstein JB, Thariat J, Bensadoun RJ, Barasch A, Murphy BA, Kolnick L et al (2012) Oral complications of cancer and cancer therapy: from cancer treatment to survivorship. CA Cancer J Clin 62(6):400–422 22972543 10.3322/caac.21157

[5] Haverman TM, Raber-Durlacher JE, Raghoebar II, Rademacher WMH, Rozema FR, Hazenberg MD et al (2020) Oral chronic graft-versus-host disease: what the general dental practitioner needs to know. J Am Dent Assoc 151(11):846–856 33121606 10.1016/j.adaj.2020.08.001

[6] Janowiak-Majeranowska A, Osowski J, Mikaszewski B, Majeranowski A (2022) Secondary oral cancer after systemic treatment of hematological malignancies and oral GVHD: a systematic review. Cancers 14(9) 10.3390/cancers14092175 PMC9102759 35565303

[7] Villa A, Kuten-Shorrer M (2023) Pathogenesis of oral toxicities associated with targeted therapy and immunotherapy. Int J Mol Sci 24(9) 10.3390/ijms24098188 PMC10179284 37175898

[8] Elad S, Zadik Y, Yarom N (2017) Oral complications of nonsurgical cancer therapies. Atlas Oral Maxillofac Surg Clin North Am 25(2):133–147 28778303 10.1016/j.cxom.2017.04.006

[9] Nederlandse Kankerregistratie (NKR) IKNL [Netherlands Cancer Registry (NCR) IKNL]. Overleving, Periode van diagnose (10 jaar), hematologische maligniteiten [Survival, Period of diagnosis (10 year), haematological malignancies] [Internet]. 2022, Aug 22 [2023, Mar 27]. Available from: https://iknl.nl/nkr-cijfers.

[10] World Medical Association Declaration of Helsinki: ethical principles for medical research involving human subjects. JAMA. 2013;310(20):2191-4. 10.1001/jama.2013.281053 24141714

[11] Aaronson NK, Ahmedzai S, Bergman B, Bullinger M, Cull A, Duez NJ et al (1993) The European organisation for research and treatment of cancer QLQ-C30: a quality-of-life instrument for use in international clinical trials in oncology. J Natl Cancer Inst 85:365–376 8433390 10.1093/jnci/85.5.365

[12] Hjermstad MJ, Bergenmar M, Bjordal K, Fisher SE, Hofmeister D, Montel S et al (2016) International field testing of the psychometric properties of an EORTC quality of life module for oral health: the EORTC QLQ-OH15. Support Care Cancer 24(9):3915–3924 27113466 10.1007/s00520-016-3216-0

[13] Slade GD (1997) Derivation and validation of a short-form oral health impact profile. Community Dent Oral Epidemiol 25(4):284–290 9332805 10.1111/j.1600-0528.1997.tb00941.x

[14] Thomson WM, van der Putten GJ, de Baat C, Ikebe K, Matsuda K, Enoki K et al (2011) Shortening the xerostomia inventory. Oral Surg Oral Med Oral Pathol Oral Radiol Endod 112(3):322–327 21684773 10.1016/j.tripleo.2011.03.024 PMC3154566

[15] Stolze J, Vlaanderen KCE, Raber-Durlacher JE, Brand HS (2020) The impact of hematological malignancies and their treatment on oral health-related quality of life as assessed by the OHIP-14: a systematic review. Odontology 108(3):511–520 31955297 10.1007/s10266-019-00479-7

[16] Silva LC, Sacono NT, Freire Mdo C, Costa LR, Batista AC, Silva GB (2015) The impact of low-level laser therapy on oral mucositis and quality of life in patients undergoing hematopoietic stem cell transplantation using the oral health impact profile and the functional assessment of cancer therapy-bone marrow transplantation questionnaires. Photomed Laser Surg 33(7):357–363 26154723 10.1089/pho.2015.3911

[17] Bezinelli LM, Eduardo FP, Neves VD, Correa L, Lopes RM, Michel-Crosato E et al (2016) Quality of life related to oral mucositis of patients undergoing haematopoietic stem cell transplantation and receiving specialised oral care with low-level laser therapy: a prospective observational study. Eur J Cancer Care 25(4):668–674 10.1111/ecc.12344 26087364

[18] Grando LJ, Mello A, Salvato L, Brancher AP, Del Moral JAG, Steffenello-Durigon G (2015) Impact of leukemia and lymphoma chemotherapy on oral cavity and quality of life. Spec Care Dentist 35(5):236–242 25963973 10.1111/scd.12113

[19] Nederfors T, Isaksson R, Mornstad H, Dahlof C (1997) Prevalence of perceived symptoms of dry mouth in an adult Swedish population--relation to age, sex and pharmacotherapy. Community Dent Oral Epidemiol 25(3):211–216 9192149 10.1111/j.1600-0528.1997.tb00928.x

[20] Johansson AK, Johansson A, Unell L, Ekback G, Ordell S, Carlsson GE (2012) Self-reported dry mouth in Swedish population samples aged 50, 65 and 75 years. Gerodontology 29(2):e107–e115 22050189 10.1111/j.1741-2358.2010.00420.x

[21] Adolfsson A, Lener F, Marklund B, Mossberg K, Cevik-Aras H (2022) Prevalence of dry mouth in adult patients in primary health care. Acta Odontol Scand 80(8):605–610 35617454 10.1080/00016357.2022.2069282

[22] Mercadante V, Jensen SB, Smith DK, Bohlke K, Bauman J, Brennan MT et al (2021) Salivary gland hypofunction and/or xerostomia induced by nonsurgical cancer therapies: ISOO/MASCC/ASCO guideline. J Clin Oncol 39(25):2825–2843 34283635 10.1200/JCO.21.01208

[23] Elad S, Raber-Durlacher JE, Brennan MT, Saunders DP, Mank AP, Zadik Y et al (2015) Basic oral care for hematology-oncology patients and hematopoietic stem cell transplantation recipients: a position paper from the joint task force of the Multinational Association of Supportive Care in Cancer/International Society of Oral Oncology (MASCC/ISOO) and the European Society for Blood and Marrow Transplantation (EBMT). Support Care Cancer 23(1):223–236 25189149 10.1007/s00520-014-2378-x PMC4328129

[24] Yong CW, Robinson A, Hong C (2022) Dental evaluation prior to cancer therapy. Front Oral Health 3:876941 35510226 10.3389/froh.2022.876941 PMC9058061

[25] van Gils T, Bouma G, Bontkes HJ, Mulder CJJ, Brand HS (2017) Self-reported oral health and xerostomia in adult patients with celiac disease versus a comparison group. Oral Surg Oral Med Oral Pathol Oral Radiol 124(2):152–156 28756881 10.1016/j.oooo.2017.05.475

