# Supplementary material for: Self-perceived oral health in hemato-oncological patients and the relation to quality of life
Source: Support Care Cancer. 2024 Sep 7;32(10):643. doi: 10.1007/s00520-024-08849-w (PMC11380634; doi:10.1007/s00520-024-08849-w)
Supplement: Supplementary file 1 — (DOCX 114 kb) [file 520_2024_8849_MOESM1_ESM.docx]

**Appendix**

Table A1. Hemato-oncological treatment history, categorized per treatment modality

| **(Former) patients**  *N = 705* | | | | | | **N** | **%** |
| --- | --- | --- | --- | --- | --- | --- | --- |
| **Received hemato-oncological treatment** | Yes | | | | | 571 | 81.0 |
|  | No (includes ‘watch-and-wait’ approach) | | | | | 127 | 18.0 |
|  | Missing | | | | | 7 | 1.0 |
| **Time since diagnosis** | 0-12 months ago | | | | | 91 | 12.2 |
|  | - 1. years ago | | | | | 270 | 38.7 |
|  | > 5 years ago | | | | | 337 | 48.3 |
| **Treated patients**  (n= 571) | | | | | | **N** | **%** |
| **Latest hemato-oncological treatment session** | Within past month | | | | | 116 | 20.3 |
|  | 1 - 3 months ago | | | | | 36 | 6.3 |
|  | 3 - 12 months ago | | | | | 70 | 12.3 |
|  | 1 - 2 years ago | | | | | 85 | 14.9 |
|  | > 2 years ago | | | | | 262 | 45.9 |
|  | Not known | | | | | 1 | 0.2 |
|  | Missing | | | | | 1 | 0.2 |
| **History of hemato-oncological treatment: occurrence of each treatment modality**  (n = 571)***** | | | | | | | |
| Grouped based on **chemotherapy modality** |  | | **N** | **%** |  | **N** | **%** |
|  | **Chemo- therapy**** |  | | | | **356** | **62.3** |
|  |  | Chemotherapy,  no radiotherapy | 258 | 45.2 |  | 142 | 24.9 |
|  |  |  |  |  | + Targeted/Immunotherapy | 116 | 20.3 |
|  |  | Chemotherapy & radiotherapy******* | 97 | 17.0 |  | 59 | 10.3 |
|  |  |  |  |  | + Targeted/Immunotherapy | 38 | 6.7 |
|  |  | Unknown | 1 | 0.2 |  | 1 | 0.2 |
| Grouped based on  **SCT modality** |  | | | | | | |
|  | **SCT** |  | | | | **221** | **38.7** |
|  |  | Autologous | 136 | 23.8 |  | 86 | 15.1 |
|  |  |  |  |  | + Targeted/Immunotherapy | 50 | 8.8 |
|  |  | Allogeneic | 65 | 11.4 |  | 57 | 10.0 |
|  |  |  |  |  | + Targeted/Immunotherapy | 8 | 1.4 |
|  |  | Both (autologous followed by allogeneic transplantation) | 18 | 3.2 |  | 16 | 2.8 |
|  |  |  |  |  | + Targeted/Immunotherapy | 2 | 0.4 |
|  |  | Unknown | 2 | 0.4 |  | 2 | 0.4 |
| Grouped based on  **targeted and/or** **immunotherapy treatment** |  | | | | | | |
|  | **Targeted therapy and/or immuno-therapy** |  | | | | **208** | **36.4** |
|  |  | No chemo-, radio-, or SCT-therapy | | | | 24 | 4.2 |
| Grouped based on  **Non-specified forms of treatment**  (Other than chemo-, radio-, SCT- or targeted/immunotherapy) |  | | | | | | |
|  | **Non-specified forms of treatment** |  | | | | **71** | **12.4** |
|  |  | Not combined with any specified forms of treatment (chemo-, radio-, SCT- and/or targeted/immunotherapy) | | | | 41 | 7.2 |

Table A2: Oral hygiene habits

|  | | **N** | **%** |
| --- | --- | --- | --- |
| **Toothbrushing** | Two times a day or more | 523 | 74,2 |
|  | Once a day | 172 | 24,4 |
|  | Multiple times a week | 6 | 0,9 |
|  | Once a week or less | 4 | 0,6 |
| **Interdental cleaning** | Once a day or more | 384 | 54,5 |
|  | Multiple times a week | 144 | 20,4 |
|  | Once a week or less | 132 | 18,7 |
|  | Does not apply due to (partial) dentures | 45 | 6,4 |
| **Use of toothpaste with fluoride** | Yes | 553 | 78,4 |
|  | No | 100 | 14,2 |
|  | Patient does not know | 52 | 7,4 |
| **Dental check-up** | Once a year or more | 621 | 88,1 |
|  | Once every two or three years | 21 | 3,0 |
|  | Irregularly | 35 | 5,0 |
|  | Only in case of dental emergency | 21 | 3,0 |
|  | Never | 7 | 1,0 |

**Figure A1: Mean scores EORTC QLQ-C30 ± 1SD, Scales & Items (N=696)**


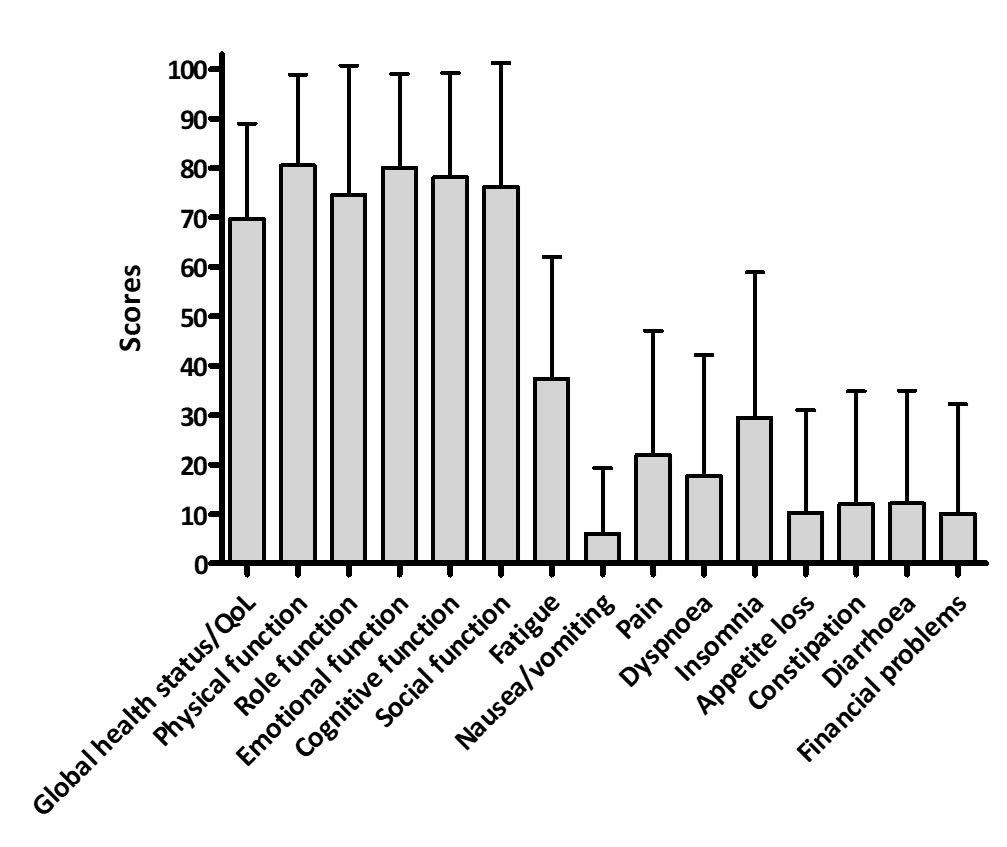


**Figure A2: percentages of rated the global health status and quality of life (EORTC QLQ-C30) (N=696)**


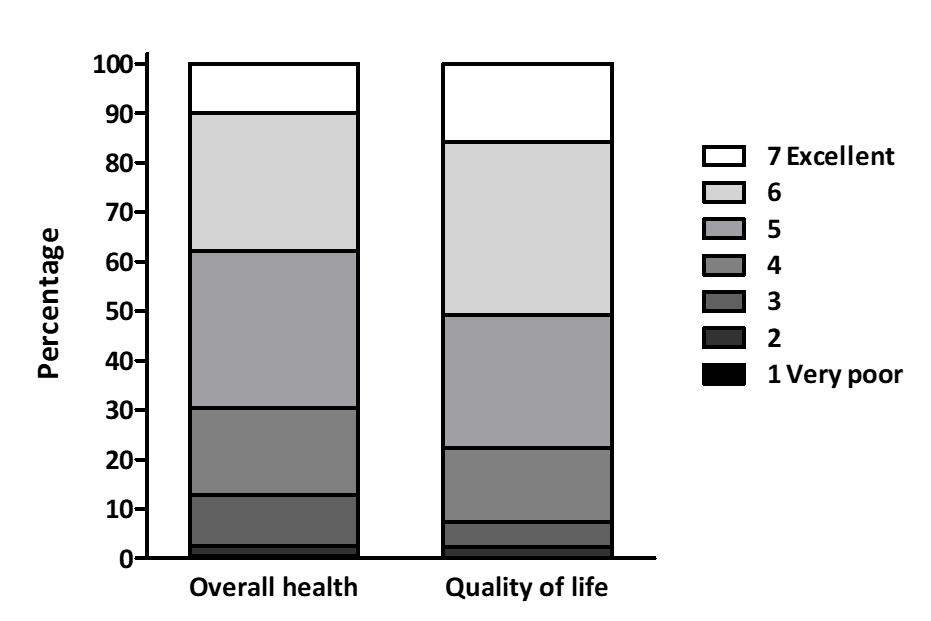


Table A3: Distribution of total scores of the shortened Xerostomia Inventory

| **Total Score** | **N** | **%** |
| --- | --- | --- |
| 5 | 202 | 28,7 |
| 6 | 111 | 15,7 |
| 7 | 95 | 13,5 |
| 8 | 90 | 12,8 |
| 9 | 71 | 10,1 |
| 10 | 52 | 7,4 |
| 11 | 25 | 3,5 |
| 12 | 20 | 2,8 |
| 13 | 12 | 1,7 |
| 14 | 8 | 1,1 |
| 15 | 8 | 1,1 |
| Missing | 11 | 1,6 |

A total score ≥ 8 qualifies as xerostomia

Table A4: OHIP-14 Highest ranked domain(s) per patient, for patients scoring > 0 on any domain (N=545)

| **Domain** | **Domain ranked as most affected by patient/highest scoring domain per patient (frequency)*** |
| --- | --- |
| Functional limitation | 52 |
| Physical disability | 34 |
| Physical pain | 387 |
| Psychological disability | 47 |
| Psychological discomfort | 174 |
| Social disability | 19 |
| Social handicap | 17 |

**Some patients reported multiple equally high scoring domains, which explains a total frequency > 545*
